# Selected Monogenic Genetic Diseases in Holstein Cattle—A Review

**DOI:** 10.3390/genes15081052

**Published:** 2024-08-10

**Authors:** Marta Gozdek, Sebastian Mucha, Adam Prostek, Tomasz Sadkowski

**Affiliations:** 1 Department of Physiological Sciences, Institute of Veterinary Medicine, Warsaw University of Life Sciences, 02-776 Warsaw, Poland; marta_gozdek@sggw.edu.pl (M.G.); adam_prostek@sggw.edu.pl (A.P.); 2 Polish Federation of Cattle Breeders and Dairy Farmers, 00-515 Warsaw, Poland; s.mucha@cgen.pl

**Keywords:** Holstein, haplotype, monogenic genetic disease, dairy cattle, fertility

## Abstract

Genetic disorders arise from alterations in the hereditary information encoded in DNA, leading to potential detrimental effects on the well-being and vitality of organisms. Within the bovine population, genetic conditions inherited in an autosomal recessive manner are frequently associated with particular breeds. In recent years, several recessive haplotypes and a few causative mutations have been discovered in Holstein cattle: CDH (Holstein cholesterol deficiency), haplotypes with a homozygous deficiency in Holstein (HH1, HH3, HH4, HH5, HH6 and HH7), BLAD (bovine leukocyte adhesion deficiency) and DUMPS (deficiency of uridine monophosphate synthase). All of these diseases are inherited in an autosomal recessive manner. From a breeding perspective, recessive mutations specifically exhibit considerable detrimental effects and are a significant problem for breeders, exposing them to economic losses. Individual mutations can cause embryo death at any stage of pregnancy. Only genetic research and conscious selection of animals for mating will lead to a reduction in the number of carriers and elimination of mutations from the population.

## 1. Introduction

**Figure 1:**
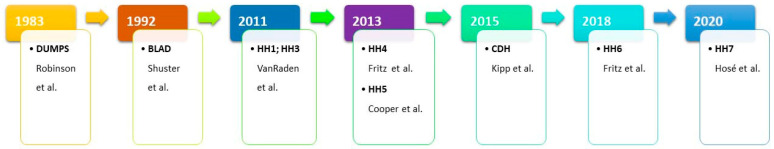
Discovery of selected genetic diseases in Holstein cattle [2,3,4,5,6,7,8,9].

In cattle breeding, genetic disorders are among the most critical points of attention for breeders. Diseases occurring during the first weeks of a calf’s life not only influence the vitality and well-being of the newborn animal but also compromise its health and productivity in later life. Calf morbidity and mortality are associated with high costs for the farmer, such as the costs of medical treatment [1]. In Holstein cattle, numerous recessive haplotypes and a limited number of causative mutations have recently been identified [2,3,4,5,6,7,8,9] (Figure 1).

Most of them affect fertility due to increased embryonic mortality. Reproduction is considered a critical attribute in the field of animal husbandry. A notable decline in the reproductive capacity of dairy cows has been documented over an extended period in numerous nations. The first-service conception rate has declined over the last 30 years from 60–70% to 30–40% [10]. Similar results have been observed in the USA, with Holstein cow conception rates near 35% [11]. The root of these problems might stem from the aggregation of genetic diseases in the population. Cattle populations are vulnerable to the spread of diseases inherited in an autosomal recessive manner. These kinds of diseases occur only in animals that inherit two mutant recessive alleles (homozygotes). Animals harboring a solitary modified allele (heterozygote) remain unaffected by the ailment yet retain the capability to transmit the genetic alterations to their progeny. Consequently, the identification of the disease in its nascent phase is considerably hindered, impeding the implementation of precautionary strategies, such as refraining from pairing affected individuals for reproduction. Most dairy cattle breeds consist of genetically small populations that originated a few centuries ago, stemming from a restricted number of founders. Over the past six decades, their genetic diversity has been further diminished due to the extensive utilization of a limited number of elite sires through artificial insemination and intense selection pressure on a narrow set of characteristics. Such intensive selection has led to an increase in inbreeding, which has translated into an increase in homozygosity and has created favorable conditions for the expression of recessive defects [5]. In cattle, autosomal recessive genetic diseases are most often breed-specific. In recent years, several recessive haplotypes and a few causative mutations have been discovered in Holstein dairy cattle: Holstein cholesterol deficiency (CDH), haplotypes with a homozygous deficiency in Holstein (HH1, HH3, HH4, HH5, HH6 and HH7), bovine leukocyte adhesion deficiency (BLAD/HHB) and deficiency of uridine monophosphate synthase (DUMPS/HHD). In this review, those marked by the Polish Federation of Cattle Breeders and Dairy Farmers for the Polish population of Holstein-Friesian cattle were selected (Table 1). All of the above diseases are somatic mutations, which means that changes in DNA happen after conception in cells other than the egg and sperm.

The aim of this review was to summarize and present available knowledge on health and reproduction in Holstein cattle.

## 2. DUMPS/HHD

Deficiency of uridine monophosphate synthase is one of the initial genetic anomalies identified in cattle (Figure 1). This anomaly is characterized as an autosomal, recessive and embryonic lethal mutation. The condition hinders the synthesis of uridine monophosphate synthase (UMPS), an enzyme pivotal in the conversion of orotic acid to uridine monophosphate (UMP), a crucial element in pyrimidine nucleotides. 

**Figure 2:**
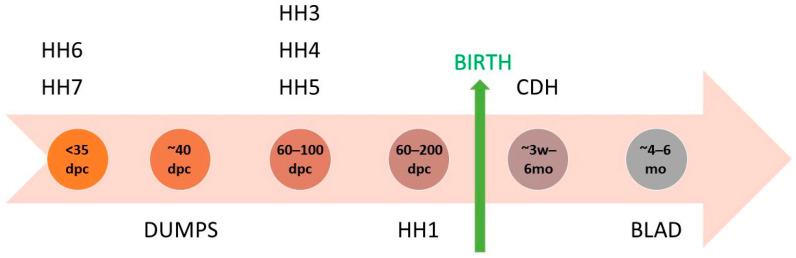
Mortality caused by the presence of deleterious mutations (homozygotes).

As nucleotides play a vital role in embryonic development, the presence of this mutation (recessive homozygote) leads to the demise of the embryo in the early stages of growth. Embryos carrying a homozygous mutation typically perish around the 40th day of gestation during uterine implantation [12] (Figure 2). 

The importance of uridine monophosphate synthase in the synthesis of DNA and RNA has generated interest in the gene encoding this enzyme [13]. The gene for UMPS is located on the long arm of chromosome 1 (BTA1, q-31–36) and consists of 1869 base pairs. Schwenger et al. [14] elucidated the underlying molecular mechanism of DUMPS, attributing it to a single nucleotide substitution in the fifth exon of the *UMPS* gene at codon 405. Specifically, the replacement of cytosine (C) by thymine (T) within the CGA codon, responsible for encoding arginine, generates a premature STOP codon TGA (Table 1) [15]. Consequently, this alteration halts the translation of the protein prematurely, leading to the loss of uridine monophosphate synthase activity.

The screening of cattle for DUMPS was formally initiated in the United States in 1988. Notably, one of the primary carriers of the DUMPS mutation was the distinguished bull Skokie Sensation Ned, who was born in the year 1957 [14]. In recent times, a significant carrier of the DUMPS mutation has been identified as the American bull named Happy Herd Beautician; the use of this bull’s semen caused the disease to spread around the world. No carrier animals were found among Holstein populations in Poland [13], Iran [16], India [17], Russia [18] and Turkey [19] (Table 2). 

## 3. BLAD/HHB

Bovine leukocyte adhesion deficiency is an autosomal recessive genetic disorder with lethal consequences, predominantly observed in Holstein cattle. It spread in Europe during the early 1990s following the beginning of the use of frozen semen. The fundamental nature of this condition lies in the compromised defensive capabilities of leukocytes. Calves possessing two copies of the mutation exhibit compromised immunity. Conversely, carriers of the BLAD mutation display full immunological functionality. Furthermore, such carriers are often prized for their significant breeding potential. Studies have indicated that cows carrying the mutation tend to produce higher quantities of milk and protein compared with their half-sisters with the standard genotype. Animals homozygous for the mutation linked to the disease suffer from deficient immunity, leading to frequent bacterial infections, oral ulcers, diarrhea and gastrointestinal inflammations that hinder proper nutrient absorption. Afflicted animals typically weigh around 60% less than their healthy counterparts [38]. These clinical manifestations result in stunted growth and development, ultimately culminating in fatality within the first few months of life due to severe infections. The root cause of these infections stems from the impaired ability of leukocytes to migrate from the bloodstream to infected tissues, attributable to the absence of a crucial membrane glycoprotein known as leukocyte integrin β-2 subunit or CD18. Integrins, as glycoproteins, play a pivotal role in mediating cell–cell and cell–substratum adhesion processes within the body, which are integral to mounting effective anti-inflammatory responses [39]. The molecular underpinnings of this malady were elucidated in 1992, revealing a point mutation in the *ITGB2* gene responsible for encoding integrin β-2 subunit [3]. Specifically, this mutation involves a substitution of adenine with guanine in the gene’s coding sequence (referred to as c.383A > G), resulting in an amino acid alteration from aspartic acid to glycine (p.Val–128Ala) (Table 1) [40]. Through the use of BovineSNP50 BeadChip, VanRaden et al. [4] confirmed the genetic locus of this disorder on bovine chromosome 1 (BTA1).

The oldest carrier of this mutation is believed to be the Osborndale Ivanhoe Friesian Holstein bull born in 1952. Bovine leukocyte adhesion deficiency carriers in Holstein cattle populations have been reported in many countries, such as China—0.48% [20]—and Turkey—between 1.3 and 2.0% [19,21]. In Mexico, Czech Republic, Russia and India, no BLAD carriers in the studied populations were found [17,18,22,23,24]. However, Ignetious et al. [25] reported BLAD at a frequency of 4% in India (Table 2). The disparity observed can be attributed to variations in the sampled populations, such as cattle from diverse geographical regions.

## 4. Haplotypes with Homozygous Deficiency in Holstein

The decrease in fertilization and normal pregnancy of dairy has become a significant problem in the Holstein breed. These problems may result from the accumulation of recessive autosomal mutations in the population that cause the spread of genetic diseases. In 2011, VanRaden et al. [4] proposed to detect them by searching a deficit in homozygous animals among the tens of thousands of individuals genotyped for genomic selection with high-density SNP beadchips (50K SNPs on the BovineSNP50 BeadChip). By utilizing genotype data obtained from a considerable number of North American Holsteins, Jerseys and Brown Swiss cattle, VanRaden et al. [4] (Figure 1) successfully pinpointed five novel recessive lethal haplotypes through the identification of common haplotypes that never manifest as homozygous in living animals. Among these haplotypes, three are exclusive to the Holstein breed, and following a proposal set forth by the Holstein Association USA, VanRaden et al. [4] (Figure 2) designated them as HH1, HH2 and HH3, with the initial H denoting Holstein and the subsequent H denoting haplotype. Studies from Denmark [41], the United States, and France [5] confirmed these results and identified additional haplotypes (among others, HH4 and HH5).

### 4.1. HH1

HH1 (OMIA 000001-9913) is an autosomal recessive inherited disease. The existence and location of HH1 was confirmed by Fritz et al. [5] (Figure 1). Next, Adams et al. [42] published the causal mutation as being a nonsense mutation (a substitution of cytosine for a thymine (c.1741C > T) on chromosome 5; BTA5). The mutation takes place in the gene encoding the apoptotic protease activating factor 1 protein (*APAF1*; p.Q579X) (Table 1). This mutation truncates 670 AA (53.7%) of the encoded APAF1 protein that contains a WD40 domain critical to protein–protein interactions. The APAF1 protein is an important cytoplasmic molecule in the cell apoptosis process and central nervous system development during embryogenesis. The necessity of functional APAF1 protein for embryo development is evident, as homozygosity for this allele naturally leads to spontaneous abortion. In Holstein cattle, the mutation causes fetal and embryonic loss between 60 and 200 days of gestation (Figure 2). The mutation has reduced conception rate and has caused an estimated 525,000 spontaneous abortions worldwide over the past 35 years and a loss of USD 420 million to the dairy industry [42]. 

HH1 traces back to bull Pawnee Farm Arlinda Chief and its descendants, i.e., Walkway Chief Mark and Millu Betty Invahoe Chief. The three bulls were important sires in the history of the Holstein cattle breed and were used for the study of the *APAF1* mutation. Research conducted by the team of [35] was intended to investigate the effects of recessive haplotypes on milk, fat and protein yields; somatic cell score (SCS); single-trait productive life (PL); daughter pregnancy rate (DPR); heifer conception rate (HCR); and cow conception rate (CCR). There were no phenotypic differences between HH1 carriers and noncarriers, but carriers had significantly lower direct genome value (DGV) for milk and protein yields, PL and fertility, as well as higher (unfavorable) DGV for SCS. VanRaden et al. [4] reported haplotype frequency as 2.25% for HH1. However, other researchers reported HH1 frequency as 1.92%, but in a report of 2018, this rate was 1.28% in the USA genomic evaluation system [26]. Studies performed in Russia indicate 2.04% of carriers in bulls and 1.83% in cows [43]; in the Italian Holstein population, the carrier frequency was 3.42% [27]; in Japan, it reached 2.9% [28]; in Uruguay, it was 4.44% [29]; and in Brazil, no carriers were detected [30]. The carrier percentages indicated 6.92% for HH1 in the Chinese Holstein cattle population [31] (Table 2).

### 4.2. HH3

The founding allele for this haplotype was traced by pedigree to the sire Gray View Skyliner. Hayes et al. [44] announced a probable causative mutation for HH3 by analyzing SNP data derived from the 1000 Bull Genomes Project. More recently, Fritz and his associates validated the deleterious impact on reproductive capacity caused by HH3 in French Holstein cattle [5]. Lethal genetic mutation HH3 is responsible for a large number of embryonic losses in Holstein cattle worldwide. HH3 was observed as a non-synonymous substitution (g.T95410507C) in the structural maintenance of the *SMC2* gene located on BTA8 (OMIA 001824-9913) (Table 1). This polymorphism changes the amino acid in position 1135 from phenylalanine to serine and causes a non-neutral, non-tolerated substitution within the NTPase domain of the encoded protein. The SMC2 protein plays a crucial function in various processes, such as DNA repair, chromosome condensation and the segregation that occurs during cellular division. An embryo is affected by this disease in a homozygous recessive system before 60 days of gestation [4]. Häfliger et al. [45] noted the absence of any homozygous carrier of the *SMC2* gene in both the present population of over 14,000 Swiss dairy cattle and any other breed of cattle encompassed within the 1000 Bull Genomes Project.

VanRaden et al. [4] reported haplotype frequency as 2.35% for HH3. Cole et al. [35] reported HH3 frequency as 2.95%, but in the report of 2018, this rate decreased to 2.64% in the USA genomic evaluation system [26]. Hozé et al. [9] showed an HH3 frequency greater than 3% in the population of French Holstein cattle. The lethal haplotype frequency was 5.1% in German cattle [32], 3.13% in Uruguay [29] and 3.0% in Kazakhstan [33] (Table 2).

### 4.3. HH4

By analyzing Illumina Bovine 50k Beadchip genotype data from 47 878 Holstein cows in the French population, a new haplotype with a significant deficit of homozygotes in live animals was identified [5]. Following the convention of naming such haplotypes, authors named the new HH4 (Figure 1). The team provided evidence for a causal mutation—a missense mutation (g.1277227A > C) in the *GART* gene (which encodes glycinamide ribonucleotide transformylase). As a consequence of this mutation, an amino acid in the building protein is changed (asparagine is replaced with threonine, p.N290T) (Table 1). GART catalyzes the first two steps of purine biosynthesis: the transfer of formyl from 10-formyltetrahydrofolate to the amino group of glycinamide ribonucleotide and the forming of glycinamide ribonucleotide and tetrahydrofolate. An embryo affected by this disease in a homozygous recessive system (two copies of the mutated gene) usually dies between 60 and 100 days of pregnancy. The effect of this mutation is a reduction of 5.8% in heifer calving rate and of 1.74% in cow calving rate [5]. HH4 carriers occur with a frequency of 3.6% in France [5], 1.26% in Germany [34] and 0.37% in the USA [35] (Table 2).

### 4.4. HH5

By using a strategy similar to that of VanRaden et al. [4], Cooper et al. [6] discovered reduced-fertility HH5 (OMIA 001941-9913) (Figure 1). HH5 is a recessive hereditary genetic defect, the homozygous carriers of which die in the early stages of fetal development. The embryo affected by this disease as a homozygous recessive dies before the 60th day of pregnancy. Schütz et al. [32] identified the likely causal mutation as the deletion of 138 kb, spanning positions 93,233 kb to 93,371 kb on chromosome 9 (BTA9), harboring only dimethyl-adenosine transferase 1 (TFB1M) (Table 1). The main function of this enzyme is to di-methylate adenine residues in the hairpin loop at the 3′ end of mitochondrial 12S rRNA. The encoded protein is also part of the basal mitochondrial transcription complex—the small subunit of mitochondria necessary for ribosomal gene expression. HH5 traces back to a bull of Canadian origin—Thornlea Texal Supreme (born in 1957). The carrier percentages indicated 4.30% for HH5 in the Chinese Holstein cattle population [31]. Schütz et al. [32] estimated the prevalence of carriers to be 5.5% in the analyzed German Holstein population, whereas in the USA, it was 2.76% [35] (Table 2). In Russia, 1202 sires and 708 Holstein cows were screened. The presence of 39 bulls and 10 cows carriers of the mutant allele of the TFB1M gene was revealed, which corresponds to the frequencies of 3.24% and 1.41%, respectively [43]. In addition, studies conducted by Cole et al. [35] showed that HH5 carriers had lower DGV for both protein yield and DPR.

### 4.5. HH6

A similar strategy to that for HH5 analysis—the homozygous haplotype deficiency strategy—was used by Fritz et al. [8], who discovered another reduced-fertility haplotype (Figure 1). In their study, by analyzing genotypes (with BovineSNP50 BeadChip) from more than 250,000 Holstein animals, the authors identified a new locus called in accordance with the accepted convention—HH6 (OMIA 002149-9913) (Table 1). Bovine embryos that are recessive homozygotes die in the first 35 days of gestation (Figure 2). Haplotype Holstein 6 is an autosomal recessive embryonic lethal mutation located on chromosome 16 (BTA16). The causal mutation was also identified—the substitution of adenine by guanine within the *SDE2* gene, coding the telomere maintenance homolog 2 protein [8]. The A > G substitution diminishes initiation codon ATG to ACG, and in effect, translation starts at the subsequent closest ATG codon, which truncates the SDE2 protein by 83 amino acids. 

The disease associated with the haplotype traces to Holstein sire MOUNTAIN, born in 1987. Since HH6 is one of the newly discovered genetic defects, there are very few reports on the frequency of carriers in Holstein cattle. Fritz et al. [8] screened 29,000 animals and found 1.3% of HH6 carriers in French Holstein cattle. Kamiński [46] reported 50 HH6 carriers among 87 Holstein bulls (57.47%). In Russia, it was found that the frequency of occurrence of carriers of HH6 in the genotyped group (60 cows and 63 bulls) was less than 1% [36] (Table 2).

### 4.6. HH7

In their study, Hozé et al. [9] analyzed the largest known panel of Illumina BovineSNP50 genotypes, comprising 401,896 Holstein animals, and reported the mapping of a new embryonic lethal haplotype called HH7, located on chromosome 27 (BTA27) (OMIA 001830-9913) (Table 1). For verification, the authors genotyped the candidate variant in 232,775 Holstein individuals and did not observe any homozygotes, whereas 16 were expected. Genotyping 250,602 animals from 19 additional breeds showed that the mutant allele is exclusively present in animals descending from the Holstein lineage. Hozé et al. [9] identified a potential causal variant associated with the novel HH7, which was characterized as a 4-base-pair deletion located in exon 11 of the gene responsible for centromere protein U (CENPU). This protein is a crucial component of the centromere, playing a vital role in ensuring accurate chromosome segregation during the process of mitosis. Bovine embryos being recessive homozygotes die within 35 days after conception (Figure 2).

The disease-associated haplotype has been traced over the last 50 years to three elite Holstein bulls: SECRET (born in 1980), MERDRIGNAC (born in 1996) and DUTCH BOY (born in 1996). This haplotype has a carrier frequency of 1.1% in the French Holstein cattle population [9] (Table 2). To our knowledge, no other reports have been published on the frequency of HH7.

## 5. CDH

Numerous calves showing incurable idiopathic diarrhea were observed in the German Holstein population in 2015. The affected calves were underdeveloped in weight and showed progressive and severe emaciation despite normal feed intake. These calves showed secondary diseases and symptoms, such as pneumonia and edema. Severe hypocholesterolemia, low triglycerides and a deficiency of fat-soluble vitamins were observed in the affected calves [7]. No obvious etiological cause of disease could be identified, and the affected calves did not respond to symptomatic medical treatment. The animals died at ages ranging from 3 weeks to 6 months [47,48]. 

In 2015, the aforementioned disease was identified as fat metabolism-linked disorder, was termed Holstein cholesterol deficiency or cholesterol deficiency haplotype (HCD; CDH; OMIA 001965-9913—Online Mendelian Inheritance in Animals database) and was reported for the first time as a new recessive defect in Holstein cattle [7]. The genome-wide association study uncovered a disease-associated haplotype on chromosome 11 (BTA11) (Table 1) [7]. The mutation represents a 1.3 kb insertion of a transposable LTR element (ERV2-1) in the coding sequence of the Apolipoprotein B gene (*APOB*), which leads to truncated transcripts. Apolipoprotein B takes part in carrying fat molecules (lipids), including cholesterol, around the body to all cells within all tissues. This finding was further supported by the RNA sequencing of the liver transcriptome of an affected calf [49] and was independently confirmed by Charlier [50] and Schütz et al. [32]. Gross et al. [51] reported that despite the presence of the *APOB* mutation, cholesterol is not limiting for animals’ metabolic adaptation and performance in heterozygous Holstein cows.

The mutation associated with Holstein cholesterol deficiency traces back to North American bull Maughlin Storm, born in 1991, whose semen was used extensively in the Holstein population worldwide [35]. Kipp et al. [7] estimated that around 3400 Holstein calves homozygous for this haplotype are born each year in Germany, giving a carrier frequency of around 8.7%. Schütz et al. [32] estimated the carrier frequency in Holstein cattle born between 2012 and 2015 in Germany to be approximately 12.5%. Wang et al. [37] estimated a carrier frequency of around 14.6% in Canadian cattle and Ussenbekov et al. [33] observed 11.0% carriers of CDH in Kazakhstan (Table 2). The information given above indicates the high prevalence of this mutation in the Holstein cattle population.

## 6. Conclusions

In recent years, several causal mutations have been identified in dairy cattle, the majority of which have a detrimental impact on fertility, leading to heightened embryo mortality (Table 1, Figure 2). All of these conditions are transmitted in an autosomal recessive pattern. From a breeding perspective, it is specifically recessive mutations that exhibit significant adverse effects. Animals carrying a single modified allele (heterozygote) do not exhibit symptoms but can transmit the mutations to their progeny. This complicates early disease detection and the implementation of preventative actions, such as refraining from breeding affected individuals and utilizing their semen for insemination. Therefore, it is crucial to conduct population screenings and pinpoint carriers to mitigate financial losses stemming from these hereditary ailments within livestock. The global rise in the use of genetic testing allows for the monitoring of the population to eradicate harmful mutations. Currently, all genetic diseases described in this article are identified by the Bovine Genetics Laboratory of the Polish Federation of Cattle Breeders and Dairy Farmers on the Illumina microarray, used for the genomic assessment of cattle around the world, as well as in Poland (within the EuroGenomics Cooperative). The working group on genetic diseases in cattle is constantly expanding the list of genetic defects and systematically adding them to the microarray.

## Figures and Tables

**Table 1:** Basic characteristics of selected genetic disorders.

Haplotype Name	Name of Disorder	Gene	OMIA	Variant Description		
				Chr	Description	Coding DNA Change
DUMPS/HHD	Deficiency of uridine monophosphate synthase	*UMPS*	000262-9913	1	SNV (nonsense)	c.1213C > T
BLAD/HHB	Bovine leukocyte adhesion deficiency	*ITGB2*	000595-9913	1	SNV (missense)	c.383A > G
HH1	Holstein haplotype 1	*APAF1*	000001-9913	5	SNV (nonsense)	c.1702C > T
HH3	Holstein haplotype 3	*SMC2*	001824-9913	8	SNV (missense)	c.3404T > C
HH4	Holstein haplotype 4	*GART*	001826–9913	1	SNV (missense)	c.869A > C
HH5	Holstein haplotype 5	*TFB1M*	001941-9913	9	Gross deletion	139 kb deletion
HH6	Holstein haplotype 6	*SDE2*	002149-9913	16	SNV (start-lost)	c.2T > C
HH7	Holstein haplotype 7	*CENPU*	001830-9913	27	5 bp deletion (splice site)	c.15123637_15123640delTTACT
CDH	Holstein cholesterol deficiency	*APOB*	001965-9913	11	Large insertion (frameshift)	ERV insertion

**Table 2:** Frequency of selected genetic diseases in Holstein cattle in different countries.

Defect	Carriers	Country	References
DUMPS	0.0%	Poland, Iran, India, Russia, Turkey	[13,16,17,18,19]
BLAD	0.48%	China	[20]
	1.3%; 2.0%	Turkey	[19,21]
	0.0% 4.0%	Mexico, Czech Republic, Russia, India India	[17,18,22,23,24] [25]
HH1	1.28%	USA	[26]
	3.42%	Italian	[27]
	2.9%	Japan	[28]
	4.44% 0.0%	Uruguay Brazil	[29] [30]
	6.92%	China	[31]
HH3	2.64%	USA	[26]
	3.0%	France	[9]
	5.1%	Germany	[32]
	3.13%	Uruguay	[29]
	3.0%	Kazakhstan	[33]
HH4	3.6%	France	[5]
	1.26%	Germany	[34]
	0.37%	USA	[35]
HH5	4.30%	China	[31]
	5.5%	Germany	[32]
	2.76%	USA	[35]
HH6	1.3%	France	[8]
	<1.0%	Russia	[36]
HH7	1.1%	France	[9]
CDH	8.7%	Germany	[32]
	14.6%	Canada	[37]
	11.0%	Kazakhstan	[33]

## Data Availability

Not applicable.
